# Serum levels of interleukin‐6 may predict organ dysfunction earlier than SOFA score

**DOI:** 10.1002/ams2.263

**Published:** 2017-03-06

**Authors:** Takashi Shimazui, Yosuke Matsumura, Taka‐aki Nakada, Shigeto Oda

**Affiliations:** ^1^ Department of Emergency and Critical Care Medicine Chiba University Graduate School of Medicine Chiba Japan

**Keywords:** C‐reactive protein, critical illness, interleukin‐6, procalcitonin, sequential organ failure assessment scores

## Abstract

**Aim:**

To investigate the clinical utility of interleukin‐6 (IL‐6), procalcitonin (PCT), and C‐reactive protein (CRP) as predictive markers in consideration of the time‐course changes in critically ill patients with organ dysfunction.

**Methods:**

Serum levels of IL‐6, PCT, CRP, and Sequential Organ Failure Assessment (SOFA) scores were measured sequentially in 92 patients during their initial 5 days following admission to the intensive care unit. Maximum values were analyzed. Patients were assigned to a low ( ≤ 8), intermediate ( > 8 and ≤ 16), or high ( > 16 and ≤ 24) SOFA score group.

**Results:**

There were significant differences in the maximum serum levels of IL‐6 and PCT among the three SOFA score groups (IL‐6, *P* < 0.0001; PCT,* P* = 0.0004). Specifically, comparisons between the groups revealed significant differences in IL‐6 levels (low versus intermediate, *P* = 0.0007; intermediate versus high, *P* = 0.0010). The probability of patients with the maximum value was greatest on day 1 (56.5%) for IL‐6, on day 1 (39.1%) or day 2 (38.0%) for PCT, on day 3 (39.1%) for CRP, and on day 1 (43.5%) for SOFA score. The median (interquartile range) peak day of IL‐6 was day 1 (1–2), which was significantly earlier than that of SOFA score at day 2 (1–3) (*P* = 0.018).

**Conclusion:**

Serum levels of IL‐6 reflected the severity of organ dysfunction in critically ill patients most accurately compared to PCT and CRP. Interleukin‐6 elevated soonest from the insult and reached its peak earlier than SOFA score.

## Introduction

Humoral mediator production by innate immunity, following recognition of a pathogen or damage‐associated molecular patterns, is a key pathophysiological component of critically ill patients with systematic inflammatory response syndrome (SIRS), such as sepsis.^1^, ^2^ Excessive production of humoral mediators, such as cytokines, can result in organ dysfunction, increasing illness severity and leading to worse outcomes in the intensive care unit (ICU).^3^ Early identification and treatment of sepsis have been the hallmark quality improvement efforts associated with improved survival in critically ill patients. The updated sepsis definition (Sepsis‐3) highlights acute organ dysfunction rather than SIRS.^4^

Interleukin‐6 (IL‐6), a well‐known cytokine that increases in the early phase of acute illness, may reflect the severity of illness or clinical outcomes in patients with SIRS.^5^, ^6^ Procalcitonin (PCT), a known biomarker of infection, is also an indicator of septic organ dysfunction.^7^ C‐reactive protein (CRP) is often measured in acute clinical settings.^8^ Investigation of biomarkers in critically ill patients with organ dysfunction may contribute to the early recognition of high‐risk patients in order to improve clinical outcomes.

Studies have indicated the utility of severity scores for acute/critical care, such as the Acute Physiology and Chronic Health Evaluation (APACHE) II score and the Sequential Organ Failure Assessment (SOFA) score.^9^ However, multiple laboratory tests and medical history are necessary to calculate severity scores, and this is a barrier to their routine utilization in the early phase of acute care. Conversely, a biomarker measurement requires a single laboratory test, which is simple and rapid. Following an insult, such as infection, biomarker levels change according to different time‐courses.^10^, ^11^ Understanding these time‐courses is essential for effective biomarker utilization. At present, there are insufficient studies on the time‐course changes of IL‐6, PCT, and CRP.

In this study, we hypothesized that serum levels of IL‐6, PCT, or CRP reflected the SOFA score in critically ill patients. We undertook serial measurements of serum levels in patients with organ dysfunction and examined differences in serum levels according to the severity of organ dysfunction, time‐course changes, and the association within each biomarker, in order to evaluate their clinical utility.

## Methods

### Patients

The current observational study was prospectively carried out. Patients admitted to the surgical/medical ICU at Chiba University Hospital (Chiba, Japan) between February 2012 and July 2013 were screened. The study included adult (≥18 years of age) critically ill patients who satisfied the following criteria consecutively during the study period: (i) ICU admission; (ii) diagnosis of SIRS according to the American College of Chest Physicians/Society of Critical Care Medicine (ACCP/SCCM)^2^ criteria on ICU admission; (iii) predicted duration of ICU stay >4 days; and (iv) new organ dysfunction according to the Brussels criteria (SOFA score ≥2)^12^ on ICU admission.

In the evaluation of the precise time‐course of the biomarker kinetics, we limited our analysis only to the patients with an obvious onset of insult (trauma, infection, cardiac arrest, acute pancreatitis, and burn) occurring within 24 h of the day 1 serum sample collection to exclude the impact of the treatment for biomarkers. We then evaluated the ratio of the biomarker values to the maximum value over the first 5 days.

### Measurements

Serum levels of IL‐6, PCT, and CRP were measured in clinical laboratories at Chiba University Hospital, as routine laboratory measurements within 2 h of collection, using rapid measurement systems (PCT and IL‐6, Roche Diagnostics, Tokyo, Japan; CRP, DENKA SEIKEN Co. Ltd., Tokyo, Japan). The measurement systems require <20 min for the measurement of IL‐6, PCT, and CRP levels. In this study, we defined the early phase of acute illness as the first 5 days from the ICU admission (day of ICU admission is day 1) and biomarker values were observed during this period. Blood samples were collected on ICU admission as a day 1 sample, then measured daily at 6 am on days 2–5 of the ICU stay. Biomarkers and SOFA score were also measured when patients survived and discharged from the ICU less than 5 days. The patients who died within 5 days in the ICU were observed and recorded as long as possible.

### Data collection and definition

The SOFA score was calculated on ICU admission and serially every day until day 5. Maximum SOFA score and maximum serum levels of IL‐6, PCT, and CRP were defined by the highest value over days 1–5. We defined the day when the SOFA score or biomarker reached its maximum value as a peak day. Patients were assigned to three groups, according to the maximum SOFA score: low SOFA (≤8), intermediate SOFA (>8 and ≤16), and high SOFA (>16 and ≤24) group. The APACHE II score was recorded on ICU admission. We assigned the Glasgow Coma Scale score at non‐sedative state to the patients who were sedated on scoring to avoid incorrectly high scores.

Sepsis was defined as the presence of SIRS induced by infection, according to the ACCP/SCCM criteria.^2^ Severe sepsis was defined as the presence of sepsis with new organ dysfunction involving at least one organ; septic shock was defined as sepsis with hypotension despite adequate fluid resuscitation, requiring vasopressor support (Sepsis‐2).^13^

### Approval of research protocol and informed consent

The protocol for this research project was approved by a suitably constituted Ethics Committee of Chiba University Graduate School of Medicine Institutional Review Board (Approval No. 1286) and conformed to the provisions of the Declaration of Helsinki. Informed consent was obtained from the subjects and/or guardians.

### Outcomes

The primary outcomes were relationship between maximum levels of biomarkers (IL‐6, PCT, and CRP) and severity of maximum SOFA score (low, intermediate, and high) during the first 5 days from ICU admission. The secondary outcomes were the peak day of SOFA score and biomarkers, time‐course changes of each biomarker from the insult, and the association among the maximum value of biomarkers.

### Statistical analysis

The relationship between maximum levels of biomarkers and the SOFA score was analyzed using the Kruskal–Wallis with Steel's post hoc test. The peak day of SOFA score and biomarkers were compared using Wilcoxon's signed‐rank test. The associations among the biomarkers were analyzed using linear regression and expressed as Pearson's correlation coefficient. Serum levels of IL‐6 and PCT were log transformed to achieve a normal distribution for this correlation analysis. Data are expressed as median (interquartile range [IQR]) for continuous variables. Two‐tailed *P*‐values <0.05 were considered significant. Analyses were carried out using JMP Pro 12 (SAS Institute Inc., Cary, NC, USA) statistical software.

## Results

**Figure 1:**
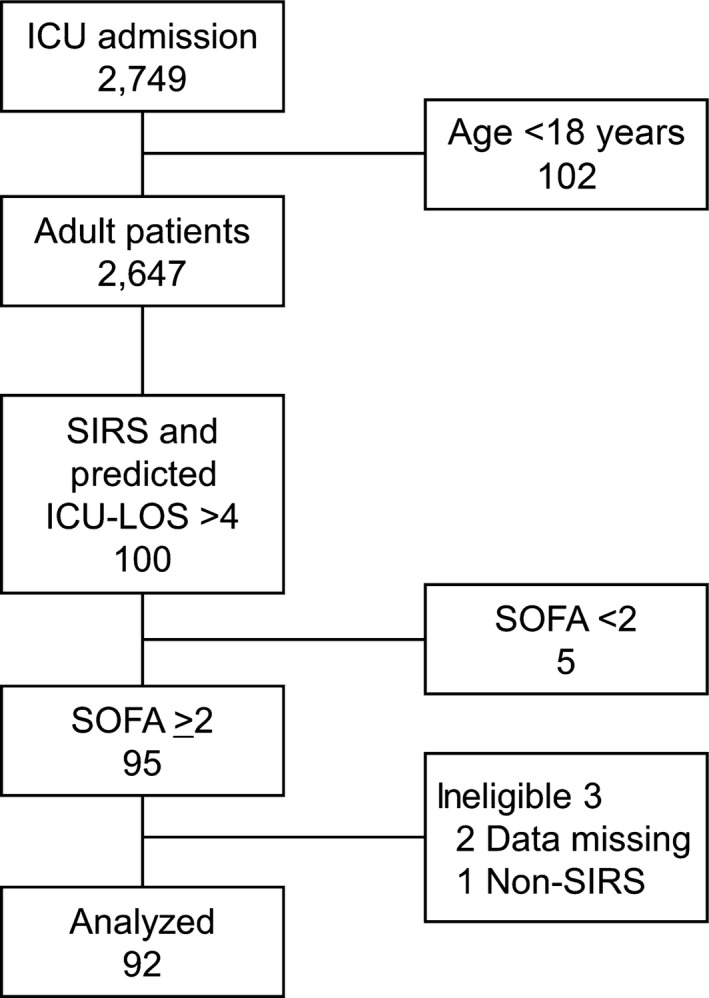
Flow diagram of patient recruitment to this study. During the study period, 2,749 patients were admitted to the intensive care unit (ICU) and 102 were excluded due to age (<18 years). One hundred patients with systematic inflammatory response syndrome (SIRS) whose predicted length of stay (LOS) in the ICU was >4 days were enrolled. Ninety five patients had a Sequential Organ Failure Assessment (SOFA) score ≥2. After exclusion of three ineligible patients, 92 patients were analyzed.

In the study period, 2,749 patients were admitted to the ICU. One hundred and two patients were excluded by their age (<18 years). Of these, 100 SIRS patients who were predicted to stay >4 days were enrolled. Of these, 95 patients presented new organ dysfunction. After exclusion of three ineligible patients (two cases had missing data, one patient without SIRS was enrolled incorrectly), 92 adult ICU patients with organ dysfunction were analyzed (Fig. 1).

The median SOFA score on admission was 10 (IQR 7–14) and 90‐day mortality was 17.4% (Table 1). Serial measurements showed that the highest levels of IL‐6, PCT, and CRP were reached on different days (day 1 for IL‐6 [953 pg/mL], day 2 for PCT [9.28 ng/mL], and day 3 for CRP [18.6 mg/dL]). The probability of patients with the maximum value was greatest on day 1 for IL‐6 (56.5%), on day 1 or 2 for PCT (39.1 or 38.0%), on day 3 for CRP (39.1%), and on day 1 for SOFA score (43.5%). The median peak day of SOFA score was day 2 (IQR 1–3), IL‐6 day 1 (IQR 1–2), PCT day 2 (IQR 1–2), and CRP day 3 (IQR 2–3). In the comparison of the peak day between SOFA score and biomarkers, IL‐6 presented with a significantly earlier peak day than SOFA score (*P* = 0.0180) (Table 2).

**Table 1:** Baseline characteristics and clinical outcomes of study participants

Characteristics or outcomes	*n* = 92
Age, years	67 (55–76)
Male, *n* (%)	69 (75.0)
APACHE II score	32 (24–37)
SOFA score day 1	10 (7–14)
Diagnosis, *n* (%)	
Infection	70 (76.1)
Severe sepsis	32 (34.8)
Septic shock	38 (41.3)
Non‐infection	22 (23.9)
Post cardiac arrest	12 (13.0)
Trauma	5 (5.4)
Acute pancreatitis	4 (4.3)
Burn	1 (1.1)
RRT within 4 days, *n* (%)	54 (58.7)
90‐day mortality, *n* (%)	16 (17.4)

Data are expressed as median (interquartile range) for continuous variables.

APACHE, Acute Physiology and Chronic Health Evaluation; RRT, renal replacement therapy; SOFA, Sequential Organ Failure Assessment.

**Table 2:** Daily values and peak day of biomarkers and Sequential Organ Failure Assessment (SOFA) score in critically ill patients with organ dysfunction

Biomarkers or SOFA score	Level/point or peak day	Patients with maximum value, *n* (%)
IL‐6		
Serum level, pg/mL		
Day 1	953 (157–9,451)	52 (56.5)
Day 2	508 (175–3,252)	22 (23.9)
Day 3	158 (87–536)	7 (7.6)
Day 4	112 (51–358)	8 (8.7)
Day 5	99 (40–182)	3 (3.3)
Maximum	2,241 (481–20,903)	
Peak day	1 (1–2)	
PCT		
Serum level, ng/mL		
Day 1	5.50 (0.37–52.90)	36 (39.1)
Day 2	9.28 (1.86–38.02)	35 (38.0)
Day 3	6.63 (2.38–33.85)	16 (17.4)
Day 4	3.90 (1.63–18.66)	3 (3.3)
Day 5	2.41 (1.10–8.35)	2 (2.2)
Maximum	11.24 (3.51–60.92)	
Peak day	2 (1–2)	
CRP		
Serum level, mg/mL		
Day 1	11.7 (2.2–25.0)	22 (23.9)
Day 2	16.4 (7.5–23.8)	14 (15.2)
Day 3	18.6 (13.0–27.4)	36 (39.1)
Day 4	16.0 (9.6–23.5)	10 (10.9)
Day 5	11.2 (7.1–19.9)	10 (10.9)
Maximum	24.3 (16.1–29.3)	
Peak day	3 (2–3)	
SOFA score		
Day 1	10 (7–14)	40 (43.5)
Day 2	10 (7–13)	22 (23.9)
Day 3	9 (6–13)	17 (18.5)
Day 4	9 (6–13)	7 (7.6)
Day 5	8 (5–13)	6 (6.5)
Maximum	13 (8–16)	
Peak day	2 (1–3)	

Data are expressed as median (interquartile range) and number (percentage).

CRP, C‐reactive protein; IL‐6, interleukin‐6; Maximum, maximum value during initial 5 days; PCT, procalcitonin; Peak day, the day when SOFA score or biomarkers reached its maximum value.

**Figure 2:**
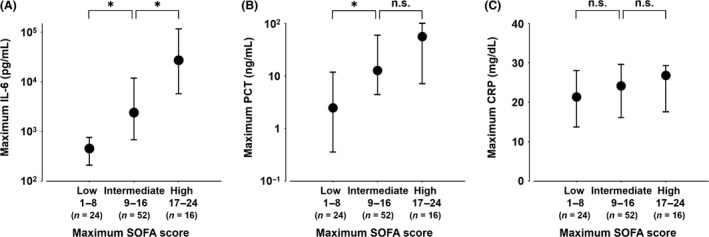
Serum levels of interleukin‐6 (IL‐6), procalcitonin (PCT), and C‐reactive protein (CRP) in critically ill patients with low, intermediate, and high Sequential Organ Failure Assessment (SOFA) scores. There were significant differences in serum levels of IL‐6 as well as PCT, but not CRP between the low, intermediate, and high SOFA score groups (low versus intermediate versus high: IL‐6, *P* < 0.0001; PCT,* P* = 0.0004; CRP,* P* = 0.28). A, IL‐6 (low versus intermediate, *P* = 0.0007; intermediate versus high, *P* = 0.0010). B, PCT (low versus intermediate, *P* = 0.0018; intermediate versus high, *P* = 0.25). C, CRP (low versus intermediate, *P* = 0.24; intermediate versus high, *P* = 0.98). Maximum serum level and SOFA score during the initial 5 days were used. Data are median and interquartile range. *P*‐values were calculated using Kruskal–Wallis with Steel's post hoc test. *P < 0.01. n.s., not significant.

For the primary analysis of the relationship between biomarkers and severity of organ dysfunction, there were significant differences in serum levels of IL‐6 and PCT among the low, intermediate, and high SOFA score group, although no difference was observed in CRP levels (low versus intermediate versus high: IL‐6, *P* < 0.0001; PCT, *P* = 0.0004; CRP, *P* = 0.28) (Fig. 2). In post hoc pairwise comparison, there were significant differences in IL‐6 levels between each group (low versus intermediate, *P* = 0.0007; intermediate versus high, *P* = 0.0010). There were significant differences in PCT levels between the low and intermediate group but not between the intermediate and high group (low versus intermediate, *P* = 0.0018; intermediate versus high, *P* = 0.25) (Fig. 2).

**Figure 3:**
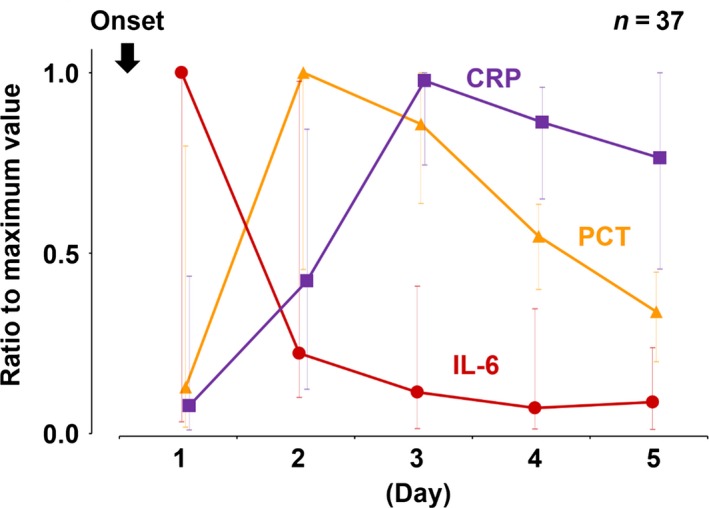
Time‐course of serum interleukin‐6 (IL‐6), procalcitonin (PCT), and C‐reactive protein (CRP) in patients with obvious insult onset. Ratio to maximum value of IL‐6, PCT, and CRP were calculated in patients with obvious insult onset and initial sample collected within 24 h after the insult. Patients had highest ratio of IL‐6 on day 1, PCT on day 2, and CRP on day 3. Data are median and interquartile range.

In the evaluation of the time‐course of the biomarker kinetics, 37 patients were chosen as patients with an obvious onset of insult. These patients had the highest ratio of IL‐6 on day 1, PCT on day 2, and CRP on day 3 (Fig. 3).

**Figure 4:**
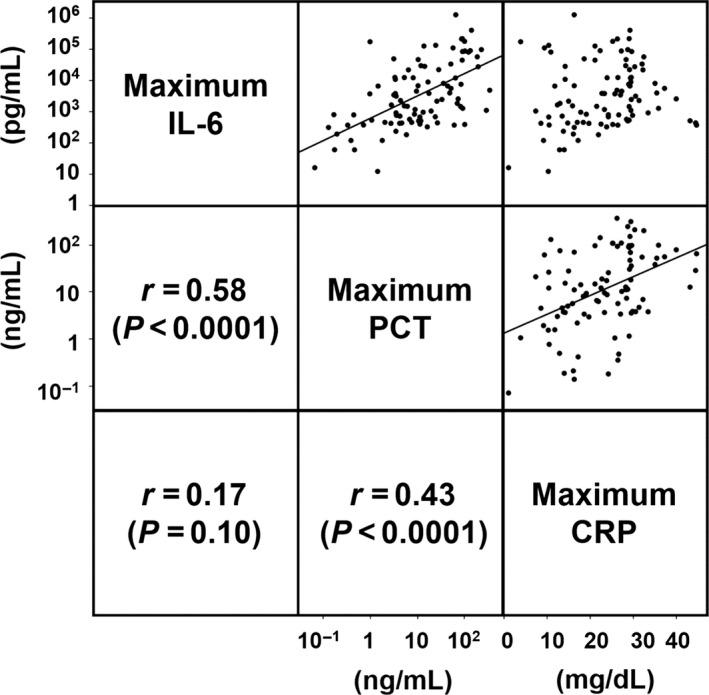
Correlation among serum interleukin‐6 (IL‐6), procalcitonin (PCT), and C‐reactive protein (CRP) in critically ill patients with organ dysfunction. Pearson correlation coefficient was calculated with the use of log‐transformed IL‐6 and PCT, and CRP.

In the association analysis of the maximum value of biomarkers, we revealed significant correlations between IL‐6 and PCT (r = 0.58, *P* < 0.0001), and between PCT and CRP (r = 0.43, *P* < 0.0001) (Fig. 4).

## Discussion

In the present study, we compared serum levels of IL‐6, PCT, and CRP, in three groups of critically ill patients, stratified according to the SOFA score for the analysis of primary outcome. Of the three biomarkers, only IL‐6 identified three different degrees of severity of organ dysfunction, according to the SOFA score. The peak day of IL‐6 was significantly earlier than SOFA score. In the analysis of time‐course changes, the highest biomarker values were frequently found on day 1 for IL‐6 and the SOFA score, day 2 for PCT, and Day 3 for CRP.

We found significant differences in IL‐6 levels according to the three severity groups (low versus intermediate versus high [*P* < 0.0001], low versus intermediate [*P* = 0.0007]; and intermediate versus high [*P =* 0.0010]). This was consistent with the positive correlations between IL‐6 levels and severity scores, such as SOFA scores and APACHE II scores, observed in critically ill patients.^5^, ^14^, ^15^ Initial levels of IL‐6 in post‐cardiac arrest patients were likely to reflect SOFA scores more closely, compared to PCT or CRP.^6^ We did not find any differences between CRP levels SOFA score‐based severity groups, which corroborates previous findings of no significant relationship between SOFA score and CRP in septic patients.^16^ In PCT levels, post hoc pairwise analysis did not reveal significant difference between the intermediate and high SOFA score groups. Procalcitonin has been used widely as a biomarker of bacterial infection.^17^ However, previous studies reported PCT was not an indicator of organ dysfunction,^18^ particularly in the severe patient group, which could explain our result. Thus, IL‐6 may more accurately identify organ dysfunction severity than PCT or CRP.

The median (IQR) peak day was day 1 (1–2) in IL‐6, day 2 (1–2) in PCT, day 3 (2–3) in CRP, and day 2 (1–3) in SOFA score. The peak day in IL‐6 was significantly earlier than that in the SOFA score. Interleukin‐6 began to increase 1 h after the insult and then reach a peak after approximately 3–6 h.^19^, ^20^ Procalcitonin started to rise 3–4 h after the insult and peaked at 24 h.^11^, ^20^ C‐reactive protein elevated at 4–6 h and peaked at 24–50 h.^11^, ^21^ Consistent with previous findings, we reaffirmed that IL‐6 is the earliest of these biomarkers to elevate and its median peak day was day 1 (1–2), which was significantly earlier than that of the SOFA score at day 2 (1–3) in the present study. Combined with the primary analysis, IL‐6 may predict severity of organ dysfunction earlier than SOFA score most accurately of the studied biomarkers. Our results suggested that identifying the peak IL‐6 reflected the severity of organ dysfunction. Therefore, sequential measurement of IL‐6 from ICU admission to its peak‐out, combined with SOFA score, would help to identify the peak accurately, and it may be a good approach in clinical practice in critically ill patients.

In the analysis of the correlations between biomarkers, PCT significantly correlated with IL‐6 and CRP. Both PCT and IL‐6 are produced by a broad range of cells,^22^, ^23^ whereas CRP is largely produced in the liver.^21^ Interleukin‐6 induces production of both PCT and CRP.^21^, ^24^ These production sites and common pathways might affect correlations.

This study has several limitations. First, the number of studied patients and enrolled etiologies were limited. We screened the patients consecutively then consequently observed five type etiologies and only 16 non‐survivors at 90 days after admission. This could be a selection bias, and we did not analyze associations between survival and biomarkers. Second, the facility at which the study was carried out has used IL‐6 and other biomarkers in the diagnosis and confirmation of treatment response of critically ill patients in daily practice. The results of biomarkers were not blinded to medical practitioners; thus, this might affect the survival outcomes and can be a diagnosis or treatment bias. Third, identification of the maximum levels and clinical utility of IL‐6 have some uncertainties. Interleukin‐6 requires sequential measurement and the level can be inhibited by immunosuppressive drugs such as steroids. Finally, renal replacement therapy may have influenced the level of biomarkers. Despite these limitations, serum IL‐6 measurement in the early phase of acute illness might be useful to assess the severity of organ dysfunction earlier than the SOFA score.

## Conclusion

Serum levels of IL‐6 reflected the severity of organ dysfunction in critically ill patients most accurately compared to PCT and CRP. Interleukin‐6 elevated soonest following the insult and reached its peak earlier than the SOFA score.

## Conflict of Interest

T.S. and Y.M. declare no conflict of interest. T.N. has received speaking honoraria from Roche Diagnostics within the past 12 months. S.O. received research funding for this study and reagents for the measurement of interleukin‐6 and procalcitonin levels from Roche Diagnostics. The funding source had no role in the design, practice, or analysis of this study.

## References

[1] Vincent JL , Opal SM , Marshall JC , Tracey KJ . Sepsis definitions: time for change. Lancet 2013; 381: 774–5. 23472921 10.1016/S0140-6736(12)61815-7 PMC4535310

[2] American College of Chest Physicians/Society of Critical Care . Medicine Consensus Conference: definitions for sepsis and organ failure and guidelines for the use of innovative therapies in sepsis. Crit. Care Med. 1992; 20: 864–74. 1597042

[3] Cohen J . The immunopathogenesis of sepsis. Nature 2002; 420: 885–91. 12490963 10.1038/nature01326

[4] Singer M , Deutschman CS , Seymour CW *et al* The third international consensus definitions for sepsis and septic shock (sepsis‐3). JAMA 2016; 315: 801–10. 26903338 10.1001/jama.2016.0287 PMC4968574

[5] Oda S , Hirasawa H , Shiga H , Nakanishi K , Matsuda K , Nakamua M . Sequential measurement of IL‐6 blood levels in patients with systemic inflammatory response syndrome (SIRS)/sepsis. Cytokine 2005; 29: 169–75. 15652449 10.1016/j.cyto.2004.10.010

[6] Bro‐Jeppesen J , Kjaergaard J , Wanscher M *et al* The inflammatory response after out‐of‐hospital cardiac arrest is not modified by targeted temperature management at 33 degrees C or 36 degrees C. Resuscitation 2014; 85: 1480–7. 25150183 10.1016/j.resuscitation.2014.08.007

[7] Meisner M , Tschaikowsky K , Palmaers T , Schmidt J . Comparison of procalcitonin (PCT) and C‐reactive protein (CRP) plasma concentrations at different SOFA scores during the course of sepsis and MODS. Crit. Care 1999; 3: 45–50. 11056723 10.1186/cc306 PMC29013

[8] Povoa P , Coelho L , Almeida E *et al* C‐reactive protein as a marker of infection in critically ill patients. Clin. Microbiol. Infect. 2005; 11: 101–8. 15679483 10.1111/j.1469-0691.2004.01044.x

[9] Vincent JL , de Mendonca A , Cantraine F *et al* Use of the SOFA score to assess the incidence of organ dysfunction/failure in intensive care units: results of a multicenter, prospective study. Working group on “sepsis‐related problems” of the European Society of Intensive Care Medicine. Crit. Care Med. 1998; 26: 1793–800. 9824069 10.1097/00003246-199811000-00016

[10] Nakada TA , Hirasawa H , Oda S , Shiga H , Matsuda K . Blood purification for hypercytokinemia. Transfus. Apher. Sci. 2006; 35: 253–64. 17092774 10.1016/j.transci.2006.06.004

[11] Preas HL 2nd , Nylen ES , Snider RH *et al* Effects of anti‐inflammatory agents on serum levels of calcitonin precursors during human experimental endotoxemia. J. Infect. Dis. 2001; 184: 373–6. 11443567 10.1086/322031

[12] Russell JA , Walley KR , Singer J *et al* Vasopressin versus norepinephrine infusion in patients with septic shock. N. Engl. J. Med. 2008; 358: 877–87. 18305265 10.1056/NEJMoa067373

[13] Levy MM , Fink MP , Marshall JC *et al* 2001 SCCM/ESICM/ACCP/ATS/SIS International Sepsis Definitions Conference. Crit. Care Med. 2003; 31: 1250–6. 12682500 10.1097/01.CCM.0000050454.01978.3B

[14] Bro‐Jeppesen J , Kjaergaard J , Stammet P *et al* Predictive value of interleukin‐6 in post‐cardiac arrest patients treated with targeted temperature management at 33 degrees C or 36 degrees C. Resuscitation 2016; 98: 1–8. 26525271 10.1016/j.resuscitation.2015.10.009

[15] Muller B , Peri G , Doni A *et al* Circulating levels of the long pentraxin PTX3 correlate with severity of infection in critically ill patients. Crit. Care Med. 2001; 29: 1404–7. 11445697 10.1097/00003246-200107000-00017

[16] Silvestre J , Povoa P , Coelho L *et al* Is C‐reactive protein a good prognostic marker in septic patients? Intensive Care Med. 2009; 35: 909–13. 19169668 10.1007/s00134-009-1402-y

[17] Wacker C , Prkno A , Brunkhorst FM , Schlattmann P . Procalcitonin as a diagnostic marker for sepsis: a systematic review and meta‐analysis. Lancet Infect. Dis. 2013; 13: 426–35. 23375419 10.1016/S1473-3099(12)70323-7

[18] Karlsson S , Heikkinen M , Pettila V *et al* Predictive value of procalcitonin decrease in patients with severe sepsis: a prospective observational study. Crit. Care 2010; 14: R205. 21078153 10.1186/cc9327 PMC3219988

[19] Gebhard F , Pfetsch H , Steinbach G , Strecker W , Kinzl L , Bruckner UB . Is interleukin 6 an early marker of injury severity following major trauma in humans? Arch. Surg. 2000; 135: 291–5. 10722030 10.1001/archsurg.135.3.291

[20] Dandona P , Nix D , Wilson MF *et al* Procalcitonin increase after endotoxin injection in normal subjects. J. Clin. Endocrinol. Metab. 1994; 79: 1605–8. 7989463 10.1210/jcem.79.6.7989463

[21] Povoa P . C‐reactive protein: a valuable marker of sepsis. Intensive Care Med. 2002; 28: 235–43. 11904651 10.1007/s00134-002-1209-6

[22] Russwurm S , Stonans I , Stonane E *et al* Procalcitonin and CGRP‐1 mrna expression in various human tissues. Shock 2001; 16: 109–12. 11508861 10.1097/00024382-200116020-00004

[23] Hunter CA , Jones SA . IL‐6 as a keystone cytokine in health and disease. Nat. Immunol. 2015; 16: 448–57. 25898198 10.1038/ni.3153

[24] Oberhoffer M , Stonans I , Russwurm S *et al* Procalcitonin expression in human peripheral blood mononuclear cells and its modulation by lipopolysaccharides and sepsis‐related cytokines in vitro. J. Lab. Clin. Med. 1999; 134: 49–55. 10402059 10.1016/s0022-2143(99)90053-7

